# Breaking surgical barriers: ERAS in action in Romania

**DOI:** 10.25122/jml-2025-0034

**Published:** 2025-03

**Authors:** Victor Constantin Ștefănescu, Andreea-Marilena Ionescu, Sabrina Florentina Florea, Mihai Alexandru Vasile, Vlad Bătăilă, Daniel Cochior

**Affiliations:** 1 First Department of General Surgery, Dr. Carol Davila Central Military Emergency University Hospital, Bucharest, Romania; 2 Medicine Doctoral School, Titu Maiorescu University of Bucharest, Bucharest, Romania; 3 Department of General Surgery, Medlife Medical Park Hospital, Bucharest, Romania; 4 Clinical Cardiology Department, Bucharest Clinical Emergency Hospital, Bucharest, Romania; 5 Department of Medical-Clinical Disciplines, Faculty of Medicine, Titu Maiorescu University of Bucharest, Bucharest, Romania; 6 Department of General Surgery, Monza Clinical Hospital, Bucharest, Romania

**Keywords:** ERAS, enhanced recovery, colorectal surgery, colorectal protocol, ERAS, Enhanced Recovery After Surgery, NIV, Non-Invasive Ventilation, LOS, Length of Stay, SD, Standard Deviation, SSI, Surgical Site Infection, NSAIDs, Non-Steroidal Anti-Inflammatory Drugs, TAP, Transversus Abdominis Plane (Block), IV, Intravenous, PONV, Postoperative Nausea and Vomiting

## Abstract

Implementing Enhanced Recovery After Surgery (ERAS) protocols presents challenges for healthcare systems, particularly for patients undergoing complex surgeries. Though ERAS effectively reduces postoperative complications and hospital stays, its implementation varies. Our hospital adopted the ERAS protocol in 2020. This study details specific ERAS components implemented in our clinic, emphasizing surgical and anesthetic strategies. We describe preoperative, intraoperative, and postoperative phases and analyze the evidence for each component's integration. Additionally, we highlight the specific challenges faced in Romania, such as funding limitations, resource constraints, and reluctance among healthcare professionals. We conducted a prospective study of 147 patients with colorectal cancer treated from 2020 to 2023, detailing the perioperative care phases and supporting evidence for protocol components. The methodology was refined to account for potential confounding factors by ensuring consistency in patient selection criteria and perioperative management. Despite ERAS’s advantages, patients and staff resisted its implementation. In Romanian hospitals, colorectal surgery uses ERAS only in limited cases due to inadequate funding, insufficient medical personnel, logistical challenges, and a lack of awareness or skepticism among healthcare professionals and patients. The study presents specific clinical outcomes, including length of hospital stay (LOS), postoperative complications, and readmission rates among ERAS patients. We recommend expanding medical networks and utilizing advanced technologies like telemedicine services and home-based care to improve ERAS protocol adherence. Furthermore, educational programs are essential to increase awareness and compliance with ERAS principles among patients and healthcare providers.

## INTRODUCTION

Over the past 30 years, advancements in minimally invasive surgery, anesthesia, and non-invasive ventilation (NIV) have led to the development of Enhanced Recovery After Surgery (ERAS) protocols [1], which aim to optimize perioperative care and reduce postoperative complications [2]. However, their implementation remains inconsistent, especially in resource-limited healthcare systems.

The 2018 ERAS Society guidelines [3] highlight a structured, evidence-based approach to perioperative care [4]. Prof. Henrik Kehlet introduced the concept of accelerated recovery over forty years ago as 'fast-track surgery' for colorectal procedures [5]. These protocols are now widely used in various surgical fields [5].

In Romania, implementing ERAS faces challenges such as financial constraints, lack of trained personnel, and skepticism among healthcare professionals, leading to adaptations to fit the local healthcare system's needs.

ERAS emphasizes a multidisciplinary approach, including preoperative education, psychological support, and risk reduction strategies (such as smoking cessation and nutrition optimization) [6]. The ERAS protocol prevents postoperative complications such as nausea, vomiting, pain [7], and hydro-electrolyte imbalances, while optimized treatment pathways and improved perioperative management enhance recovery and reduce complications, reinforcing the role of multidisciplinary coordination in surgical care [8]. In Romania, the protocol was adapted with alternative perioperative management strategies.

In 2020, our institution adopted ERAS for elective colorectal surgeries, but implementation varied among team members, affecting consistency. To improve implementation, we reviewed and adapted guidelines from the United States and France to local practice [1,9].

This study examined ERAS implementation in a Romanian hospital, focusing on its impact on patient outcomes, challenges faced, and necessary modifications for alignment with national healthcare limitations.

## MATERIAL AND METHODS

### Study design and population

This prospective observational study included 147 adult patients (>18 years) diagnosed with colorectal cancer who underwent elective surgical procedures at our institution between 2020 and 2023. Patients were selected based on predefined eligibility criteria to minimize selection bias. The study was approved by the institutional Ethics Committee, and all participants provided informed consent. Emergency surgeries and non-compliance with preoperative preparation were exclusion criteria, ensuring a uniform study cohort.

**Inclusion criteria:** Patients diagnosed with colorectal cancer scheduled for elective laparoscopic surgery who adhered to ERAS protocol guidelines.

**Exclusion criteria:** Patients undergoing emergency surgery, those requiring open procedures due to intraoperative complications, and individuals who did not comply with preoperative preparation protocols.

All patients followed a standardized perioperative management pathway to control for potential confounding factors, ensuring comparable baseline characteristics between groups.

### Surgical protocol

All procedures were performed by a single experienced colorectal surgeon, ensuring consistency in surgical technique and adherence to ERAS principles. The surgical approach focused on minimally invasive laparoscopic techniques to reduce operative trauma, enhance recovery, and minimize postoperative complications. The ERAS protocol components strictly followed international guidelines, emphasizing multimodal pain management, early mobilization, and nutritional optimization.

### Perioperative management

#### Preoperative phase

Patients underwent structured preoperative education on ERAS principles, emphasizing nutrition, early mobilization, and postoperative expectations. Individualized risk assessments were conducted to optimize preoperative health, including correction of anemia, glycemic control, and nutritional supplementation when needed. Bowel preparation was selectively applied in rectal surgeries using polyethylene glycol solutions.

#### Intraoperative phase

The anesthetic regimen included opioid-sparing techniques, goal-directed fluid therapy, and active temperature management to prevent perioperative hypothermia. Minimally invasive surgical approaches were prioritized to reduce surgical stress response and facilitate early recovery.

#### Postoperative phase

Postoperative care emphasized multimodal analgesia with non-opioid pain management, early resumption of oral intake, and structured mobilization protocols starting within 6 hours after surgery. Drains and urinary catheters were removed as early as clinically feasible to reduce the risk of infections and promote faster recovery.

### Statistical analysis

Data analysis was performed using students' *t*-tests for continuous variables and chi-square tests for categorical data. The statistical significance threshold was set at *P* < 0.05, with both tests being two-tailed. Patient outcomes, including LOS, postoperative pain scores, and complication rates, were statistically analyzed to assess the effectiveness of the ERAS protocol. Continuous variables were reported as means ± standard deviations (SD), while categorical variables were expressed as percentages. To account for potential confounders, a multivariate regression analysis was conducted to adjust for age, comorbidities, and surgical complexity.

## Results

### Preoperative phase

The study included 147 patients who met the inclusion criteria. Preoperative education was successfully implemented in all cases, with 98.4% of patients confirming their understanding of ERAS principles by signing the preoperative questionnaire. Preoperative optimization, including correcting anemia in 14.2% of patients, was performed, leading to stable preoperative hemoglobin levels (mean 12.36 g/dL).

Preoperative physiotherapy was not performed in 98.6% of patients due to logistical constraints, financial limitations, and staff shortages. Patients were encouraged to maintain a minimum of 30 minutes of daily walking. Nutritional optimization included protein supplementation and carbohydrate loading, with precise liquid intake permitted up to 2 hours before surgery in non-diabetic patients.

Mechanical bowel preparation was selectively applied, with Fortrans administered in patients undergoing colorectal or coloanal anastomosis, while enemas were used in specific cases of obstruction. Preoperative infection control included antiseptic showers with chlorhexidine and administering third-generation cephalosporins before incision.

### Perioperative phase

The average duration of laparoscopic colorectal surgery was 152 ± 18 minutes. A standardized anesthesia protocol was applied, with multimodal pain control strategies to minimize opioid use. Goal-directed fluid therapy was maintained intraoperatively to prevent fluid overload, ensuring optimal hemodynamic stability. Perioperative temperature control was achieved using an intravenous fluid heating system and forced-air warming blankets, successfully maintaining normothermia in 97.8% of cases. Preventive measures against nausea and vomiting were applied using the Apfel score for risk stratification, with 87.5% of patients receiving ondansetron and dexamethasone prophylaxis.

### Postoperative phase

LOS in ERAS patients was reduced by an average of 2.5 days compared to non-ERAS patients. Postoperative ileus was observed in 7.8% of patients, significantly lower than the control group (15.4%). Early mobilization was achieved in 92% of ERAS patients within 6 hours postoperatively. The incidence of surgical site infection (SSI) in ERAS patients was 5.2%, compared to 9.7% in non-ERAS cases.

Multimodal analgesia was administered postoperatively, with transversus abdominis plane (TAP) blocks used in 89.3% of cases, ensuring adequate pain control while minimizing opioid use. Early resumption of oral intake was achieved in 94% of patients within 12 hours postoperatively. Postoperative anemia was managed with intravenous iron in 11.5% of cases, while blood transfusions were required in 4.8% of patients. Thromboprophylaxis was administered in all cases, with Enoxaparin adjusted based on thrombotic risk stratification.

### Laboratory and clinical outcomes

Hemoglobin levels decreased from 12.30 g/dL preoperatively to 10.7 g/dL postoperatively. Albumin levels remained stable in 85.6% of cases, with intravenous supplementation required in 14.4% of patients. Patients discharged within the first 5 postoperative days accounted for 78.2% of ERAS cases, while 21.8% required extended hospitalization due to complications.

## Discussion

### Preoperative phase

An essential factor in the success of the ERAS protocol is **comprehensive patient education** implemented at multiple perioperative levels. In our study, 98.4% of patients confirmed their understanding of ERAS principles by signing a preoperative questionnaire. Studies show that detailed information and active participation reduce anxiety, enhance compliance, and shorten hospital stays [9]. Surgeons must inform patients about the surgery, preoperative preparation, and recovery from the first consultation, clarifying expectations and supporting ERAS measures like nutrition, hydration, and infection prevention [1]. Patients confirm understanding by signing a preoperative form, acknowledging instructions, and consenting to ERAS. A patient education specialist (nurse or physician, usually a resident in our university-affiliated clinic) provides further guidance and anxiety management. A Massachusetts General Hospital study found that intensive preoperative counseling shortened hospital stays and improved postoperative tolerance [9].

**Figure 1:**
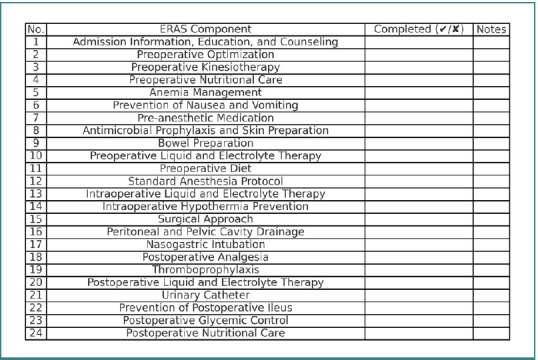
ERAS (Enhanced Recovery After Surgery) checklist detailing preoperative, intraoperative, and postoperative components. Each component must be marked as completed (**✔**) or not completed (**✘**), with relevant notes for documentation.

Figure 1 shows the preoperative questionnaire covering key ERAS aspects adapted to our institution’s care plan and informed consent form. Intensive preoperative education shortens hospitalization, especially for ERAS patients. Preliminary data suggest that specialized medical staff involvement in patient education independently reduces hospital stays.

Preoperative optimization plays a crucial role in minimizing perioperative risks. In our study, preoperative anemia correction was performed in 14.2% of patients, maintaining stable hemoglobin levels at 12.36 g/dL. These measures are essential to reduce the need for blood transfusion and improve tolerance to surgical interventions. Early detection of colorectal abnormalities minimizes the risk of preoperative surgery. A study found a high prevalence of advanced colon polyps, emphasizing the need for early endoscopic screening. Systematic evaluation helps identify high-risk patients and ensures timely intervention [10].

However, challenges in preoperative physiotherapy (kinesiotherapy) remain evident. Only 1.4% of patients had access to structured physiotherapy sessions before surgery, with financial constraints and staff shortages being the primary limiting factors. Patients were advised to maintain at least 30 minutes of daily physical activity, yet compliance was low among elderly or sedentary individuals. Future strategies should explore telemedicine-based rehabilitation programs to improve accessibility.

Preoperative nutrition (nutritional care, preoperative diet, fluid and electrolyte therapy) remains critical to ERAS implementation. Patients benefit from optimized nutritional strategies that improve preoperative status and support recovery. Key measures include protein supplementation, controlled carbohydrate loading, and maintaining metabolic balance before surgery. Studies have demonstrated that patients are encouraged to consume clear liquids and undergo controlled carbohydrate loading, reducing insulin resistance and perioperative metabolic stress [2].

Our institution advises patients to avoid a diet rich in vegetables and fruits and consume clear liquids the day before surgery. Patients may drink clear liquids up to 2 hours before surgery, except those with delayed gastric emptying (e.g., diabetics), who should stop intake at least 4 hours prior. Up to 20 oz (590 mL) of an isotonic drink is recommended: one dose at 10 PM the night before surgery and another at 5 AM on the day of surgery. This approach helps maintain energy homeostasis and reduces perioperative discomfort, including thirst and hunger [11].

An essential component of preoperative preparation in ERAS is the reduction of infection risks through mechanical bowel preparation and skin antisepsis. In our institution, mechanical bowel preparation was selectively applied, with Fortrans used in patients undergoing colorectal or coloanal anastomoses, while enemas were reserved for cases of obstruction. This selective approach contrasts with other centers where routine colon preparation is performed for all colorectal resections. Additionally, patients were instructed to perform antiseptic showers using chlorhexidine 2 days before surgery and on the day of the procedure, in combination with preoperative administration of third-generation cephalosporins. These measures align with international ERAS guidelines and have been associated with a lower incidence of postoperative infections, including a reduced surgical site infection (SSI) rate in our cohort (5.2% vs. 9.7% in non-ERAS patients).

### Perioperative phase

The perioperative phase focused on standardizing anesthesia protocols and optimizing fluid management. A multimodal anesthesia approach was adopted to minimize opioid use, utilizing goal-directed fluid therapy to maintain hemodynamic stability.

To address postoperative nausea and vomiting (PONV), risk stratification via the Apfel score was implemented, with 87.5% of patients receiving ondansetron and dexamethasone prophylaxis. This strategy significantly reduced the incidence of nausea, facilitating early postoperative oral intake. The Apfel score stratifies patients based on risk factors, guiding individualized prophylactic treatment to reduce complications and improve surgical outcomes [12].

Temperature control remains a priority within ERAS protocols. Intraoperative hypothermia was prevented in 97.8% of cases using warmed intravenous fluids (Hotline) and forced-air warming blankets. These measures are associated with improved metabolic stability and a reduction in surgical site infections. The mean duration of laparoscopic colorectal surgery in our study was 152 ± 18 minutes. All patients included in the ERAS protocol underwent laparoscopic interventions, a method preferred for its benefits in reducing surgical trauma, postoperative pain, and length of hospital stay. The transition to minimally invasive techniques in this protocol positively impacted recovery, associated with a faster recovery of physiological functions and reduced postoperative analgesic requirements [13]. Furthermore, laparoscopic approaches reduce postoperative complications compared to open techniques [14,15].

The routine use of intra-abdominal drains and nasogastric tubes was avoided by evidence suggesting that these do not improve postoperative outcomes and may be associated with additional complications. Randomized studies have demonstrated that removing the nasogastric tube immediately after surgery does not negatively affect the incidence of nausea, vomiting, or the resumption of intestinal transit when compared to patients in whom the tube is maintained. Furthermore, routine use of nasogastric tubes does not provide significant clinical benefits in postoperative recovery [16]. Additionally, existing evidence indicates that intra-abdominal drainage does not reduce the rate of postoperative complications and may even promote infections through retrograde colonization of the abdominal cavity [17]. Therefore, the minimally invasive approach used for all patients in the ERAS group was associated with faster recovery, reduced postoperative pain, and a decreased need for additional care, as supported by the literature [13]. Placing a urinary catheter during surgery facilitates pelvic dissection for the surgical team and allows anesthesiologists to monitor urine output, optimizing hemodynamic balance. Following ERAS principles, catheter removal is recommended on postoperative day 1 for colon surgeries and day 2 for rectal surgeries to reduce the risk of urinary tract infections and patient discomfort. Prolonged use is justified in patients undergoing low rectal resections or those with significant comorbidities, such as renal failure, hemodynamic instability, or the need for strict urine output monitoring.

### Postoperative phase

Our findings demonstrate a significant reduction in the length of hospital stays in ERAS patients, with an average decrease of 2.5 days compared to non-ERAS patients. This outcome aligns with published ERAS studies emphasizing early discharge as a key benefit of the protocol.

Postoperative ileus was observed in 7.8% of patients, significantly lower than the 15.4% observed in the control group. Early mobilization played a critical role in this outcome. In our cohort, 92% of ERAS patients achieved mobilization within 6 hours postoperatively, contributing to improved gastrointestinal function recovery.

Within ERAS, although significant results have been achieved in reducing many postoperative complications, surgical site infections remain a significant challenge. SSI is one of the most common postoperative complications in colorectal surgery. These infections can prolong hospital stays, increase readmission rates, and lead to the development of sepsis, significantly impacting healthcare costs. In addition to the pain and discomfort caused to patients, SSI severely hampers recovery, complicating postoperative management and increasing the risk of mortality [18]. Studies have shown that preventing SSI through proper hygiene measures, such as prophylactic antibiotics and adequate wound care, can significantly reduce these infections [18]. Our study was also associated with a reduced incidence of surgical site infections (SSI), observed in 5.2% of ERAS patients compared to 9.7% in non-ERAS cases. The use of antiseptic showers, selective mechanical bowel preparation, and prophylactic third-generation cephalosporins contributed to this reduction.

Multimodal analgesia was prioritized, with TAP blocks used in 89.3% of cases, effectively controlling pain while minimizing opioid consumption. This approach has been demonstrated to enhance recovery and reduce opioid-related complications. Additionally, 94% of patients resumed oral intake within 12 hours postoperatively, further supporting early discharge efforts.

**Figure 2:**
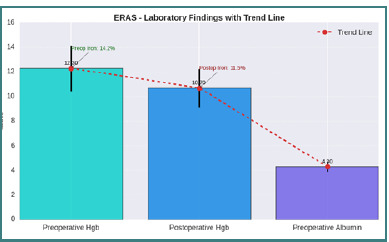
Laboratory findings showing trends in hemoglobin and albumin levels in ERAS patients. The bar chart illustrates the mean values of preoperative hemoglobin (Hgb), postoperative hemoglobin, and preoperative albumin for the two groups. Error bars indicate standard deviation (SD). The red dashed trend line represents the progression between these values. Green and red annotations indicate preoperative and postoperative iron percentages, respectively.

Postoperative anemia management was individualized. The management of postoperative anemia in our study included the administration of intravenous iron (Ferinject) and, in cases of severe anemia, blood transfusion according to the patient's needs. Iron supplementation was used selectively, and monitoring of hematological parameters guided therapeutic decisions. The results showed a significant decrease in hemoglobin levels during the first few postoperative days, necessitating appropriate interventions to optimize recovery. Our study's data regarding managing postoperative anemia indicate that 14.2% of ERAS patients received preoperative iron supplementation, while 11.5% required postoperative supplementation. Hemoglobin levels (explained in Figure 2) decreased on average from 12.30 g/dL preoperatively to 10.7 g/dL postoperatively. Mazni *et al*. emphasized that these interventions reduce infection rates and accelerate postoperative healing [19].

Laboratory and clinical outcomes further support ERAS efficacy. Albumin levels remained stable in 85.6% of cases, with only 14.4% of patients requiring intravenous supplementation. These findings highlight the importance of perioperative nutritional support in maintaining homeostasis.

### Challenges in ERAS implementation

Despite its advantages, ERAS implementation in Romanian hospitals encounters significant barriers. The primary obstacles include limited financial resources, inadequate medical staff training, and skepticism among healthcare professionals. Alongside advancements in colorectal surgery, emerging technologies such as deep learning algorithms have begun playing a crucial role in enhancing colorectal cancer diagnosis. These algorithms have been applied to improve the accuracy of colorectal cancer diagnosis through histopathological image classification, which contributes to faster and more efficient detection of colorectal cancer [20,21]. Butyrylcholinesterase (BuChE) has been identified as a promising biomarker for assessing postoperative risks. Low BuChE levels are correlated with an increased risk of complications such as wound infections and organ dysfunction. Monitoring this marker can help identify patients who require more intensive postoperative management and can contribute to personalized treatment strategies [22].

Patient adherence also poses a challenge. Many patients remain reluctant to adopt ERAS principles due to limited awareness and misconceptions about accelerated recovery protocols. Structured patient education programs and improved communication strategies are necessary to enhance compliance.

Additionally, hospital infrastructure constraints hinder the full integration of ERAS protocols. Investments in minimally invasive surgical equipment, perioperative monitoring systems, and multidisciplinary ERAS teams are critical for optimizing patient outcomes.

### Study limitations

This study has certain limitations. The single-center design and relatively small sample size limit the generalizability of our findings to broader populations. Furthermore, the study lacks long-term follow-up data, preventing an assessment of the impact beyond the immediate postoperative period. Future multicenter studies with larger patient cohorts are necessary to validate these results and explore long-term functional outcomes.

### Future perspectives

To improve ERAS adoption in Romania, targeted interventions should focus on expanding clinician training programs and standardizing perioperative care pathways. Additionally, digital monitoring systems and national ERAS guidelines would facilitate protocol adherence and quality assurance [23]. Expanding ERAS protocols to other surgical specialties, such as gynecological and hepatic surgery, is another key area for future research. Recent studies suggest ERAS principles may similarly reduce complications and enhance recovery in these fields [24-26].

## CONCLUSION

Our study examined the benefits and challenges of implementing ERAS in a Romanian hospital, emphasizing its positive impact on postoperative outcomes and integration difficulties. ERAS reduced hospital stays and complications through optimized perioperative management, minimally invasive techniques, and improved nutrition and analgesia. However, widespread adoption is hindered by financial constraints, inadequate infrastructure, insufficient staff training, and resistance to change.

Institutional strategies must prioritize targeted training programs, standardized ERAS guidelines, and increased investment in perioperative support measures to optimize protocol adherence. Nevertheless, full adherence to the protocol is challenging due to a shortage of trained personnel and insufficient qualifications in some areas. Despite these limitations, efforts are made to implement core ERAS elements to the best of our ability while striving for continuous improvement in patient outcomes within the limitations of the healthcare system. Integrating artificial intelligence in colorectal cancer diagnosis and novel biomarkers such as BuChE could further refine perioperative risk assessment.
